# Extramedullary Plasmacytoma Mimicking Pancreatic Cancer: An Unusual Presentation

**DOI:** 10.1155/2016/6919210

**Published:** 2016-10-25

**Authors:** Daniela Sciancalepore, Sergio Musci, Maria Rosaria Fracella, Grazia D'Alesio, Azzurra Sportelli, Giuseppe Ingravallo, Angelo Vacca, Roberto Ria

**Affiliations:** ^1^Department of Biomedical Sciences and Human Oncology, Section of Internal Medicine and Clinical Oncology, University of Bari, Bari, Italy; ^2^Cardiology Unit, San Paolo Hospital, Contrada Caposcardicchio, Bari, Italy; ^3^Radiology Unit, San Paolo Hospital, Contrada Caposcardicchio, Bari, Italy; ^4^Department of Pathology, University of Bari, Bari, Italy

## Abstract

Multiple myeloma is a plasma cell tumor that homes to and expands in the bone marrow and that, despite the new available drugs, remains incurable. Extramedullary plasmacytoma is a not frequent manifestation during the natural history of multiple myeloma and is frequently associated with plasma cell bone marrow infiltration. The most common locations for an EMP include the gastrointestinal tract, pleura, testis, skin, peritoneum, liver, endocrine glands, and lymph nodes. Primary involvement of the gallbladder fossa is exceedingly rare. In this report, we describe a patient with multiple myeloma who achieved a clinical and serological remission after autologous transplant but progressed rapidly at extramedullary site mimicking a second cancer (i.e., pancreatic or biliary cancer). In this case, the extramedullary localization was refractory to standard therapy, differently from bone marrow localization, but responded to lymphoma-like therapy. In this patient (i) the particular site of developing plasmacytoma is the gallbladder fossa, (ii) the timing of onset of this neoplasm is immediately after autologous transplant, and (iii) its disjunction from primary myeloma is that it appears in clinical and serological remission phase which may be confounding during the diagnostic approach simulating a different tumor (solid tumor).

## 1. Introduction

Extramedullary plasmacytomas (EMP) represent a rare manifestation in the course of multiple myeloma (MM) [1]. During the last years, however, their incidence has increased predominately in patients who undergo bone marrow transplantation due to the selection of resistant clones after intensified therapy [1] and in patients who receive thalidomide-containing regimens, probably due to dedifferentiation of bone marrow plasma cells or alterations in the expression of adhesion molecules [2].

Moreover, extramedullary progression of MM has consistently been associated with a poorer disease prognosis [3]. This poorer prognosis is not clearly related to the type or intensity of prior treatments and cannot always be explained by occurrence of this progression in very advanced disease stages. There is increasing evidence that extramedullary relapse is associated with secondary changes in the myeloma clone, aggressive disease progression, poor prognostic histological and biological factors (plasmablastic morphology, higher proliferative index, and p53 expression), and treatment resistance [4, 5].

It has also been reported that extramedullary progression or relapse is often associated with the “escape” phenomenon of the monoclonal component [6].

## 2. Case Presentation

**Figure 1:**
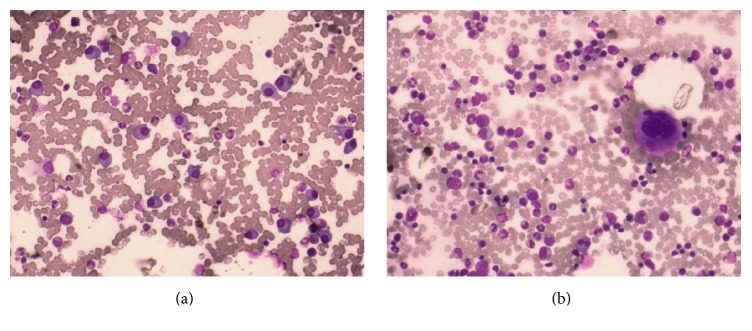
(a) May-Grunwald-Giemsa stain of bone marrow aspirate at diagnosis showing infiltrate of atypical plasma cells and (b) May-Grunwald-Giemsa stain after ASCT at VGPR showing reduction of the infiltration.

A 62-year-old man had come to our observation in December 2013 because of the onset of a monoclonal component (MC) of about 51 gr/L typing as IgG-k at immunofixation in absence of anamnestic evidence of hematologic and extrahematologic diseases. Blood tests were normal: Hb 131 g/L, platelets 176000/*μ*L, and leucocytes 5400/*μ*L. Leucocyte differential count, calcium, and LDH serum level were normal. *β*2-Microglobulin was 4.47 mg/L; albumin was 37 g/L; k/*λ* free light chain ratio was 29.03. No Bence Jones proteinuria was detected. Bone marrow plasma cell infiltration was 38% (Figure 1(a)). Skeleton X-rays and spine MRI did not visualise osteolyses. The physical examination was negative for objective evidence of disease but the patient complains of vertigo, unsteadiness in walking, muscle pain, paresthesias. The fundus oculi examination showed papilloedema. Patient was diagnosed as “*symptomatic multiple myeloma IgG-k D&S IIA, ISS 2 with hyperviscosity syndrome*” and has undergone first-line therapy consisting of induction therapy with bortezomib, thalidomide, and dexamethasone (VTD) per 4 cycles, stem cell mobilization with cyclophosphamide 4 gr/m^2^ and G-CSF, and autologous stem cell transplant (ASCT) with melphalan 200 mg/m^2^. At the evaluation of response after ASCT the patient achieved a very good partial response (VGPR); patient refused the second ASCT and consolidation therapy was started with the VTD schema.

In November 2014, after the first consolidation cycle, patient developed fever, mild abdominal pain, and jaundice. The new evaluation of myeloma showed persistence of response with MC of 3 gr/L, Hb 12 g/L, platelets 195000/*μ*L, and leucocytes 8140/*μ*L. Leucocyte differential count, *β*2-microglobulin, calcium, and LDH serum level were normal. Albumin was 30 g/L and k/*λ* free light chain ratio was 4.41, with 2% bone marrow plasma cell infiltration (Figure 1(b)) and absence of Bence Jones proteinuria as well as osteolyses at skeleton X-rays and spine MRI. Total bilirubin was of 11.9 mg/dL and direct bilirubin was of 10.03 mg/dL. Gamma-glutamyl transferase was of 892 U/L, alkaline phosphatase was of 405 U/L, alanine aminotransferase was of 51 U/L, aspartate aminotransferase was of 119 U/L, pancreatic amylase was of 130 U/L, lipase was of 2675 U/L, and CA19.9 was of 996.9 U/mL.

**Figure 2:**
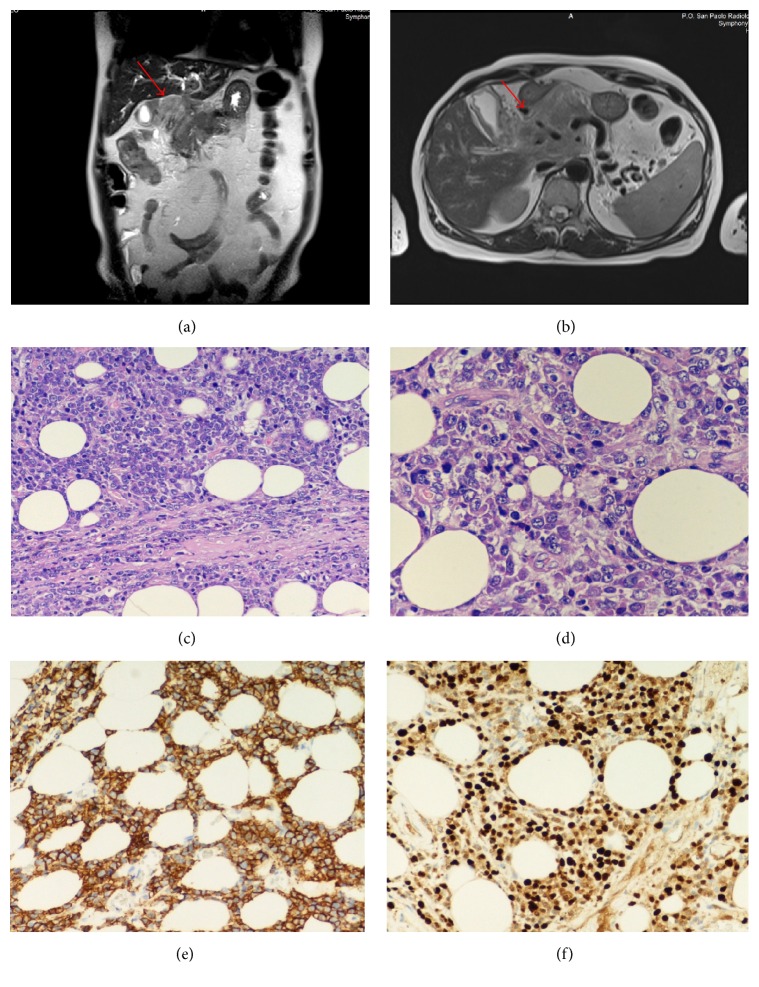
MR cholangiopancreatography before VBD therapy. (a) and (b) show a tumor mass localized at the hepatic hilum without cleavage plane with the head of the pancreas and blood vessels of about 8.35 × 8.7 × 8.9 cm. ((c) and (d)) Hematoxylin and eosin stain. Original magnification ×100 and ×200. The myeloma cell size and nucleus are polymorphic. Many cells have large eccentric nuclei, prominent nucleoli, and abundant basophilic cytoplasm (i.e., nuclear and cytoplasmic maturation asynchrony). (e) The neoplastic cells are positive for CD138. (f) The ki67 labeling index exceeds 50%.

MR cholangiopancreatography showed a tumor mass localized at the hepatic hilum without cleavage plane with the head of the pancreas and blood vessels of about 8.35 × 8.7 × 8.9 cm (Figures 2(a) and 2(b)). The site of tumor mass and its occurrence during the remission phase of myeloma, immediately after autologous transplant, made us think of a second cancer, a solid cancer starting from pancreas or extrahepatic biliary tract, so, for that suspicion, the patient was referred to surgeon for biopsy. Histological examination evidenced massive plasmoblastic localization (Figures 2(c) and 2(d)) that, at immunohistochemical staining, was positive for kappa light chain, CD138 (Figure 2(e)), and CD79a, with KI67 > 50% (Figure 2(f)). These histological findings are indicative of the selection of a clone resistant to standard myeloma therapy whose behaviour is similar to an aggressive lymphoma that quickly affected extramedullary tissues.

During the diagnostic period, since the patient had discontinued the specific therapy for myeloma for about one month, it has highlighted also a serological progression of disease with increase of the MC to 24 g/L, decrease of Hb (100 g/L) and albumin (17 g/L), alteration of k/*λ* free light chain ratio (34.12), and increase of bone marrow plasma cell infiltration to 30%.

Final diagnosis was “multiple myeloma progression with associated extramedullary plasmocytoma” and the patient underwent new therapy with bendamustine, prednisone, and lenalidomide (BPR) in December 2014.

**Figure 3:**
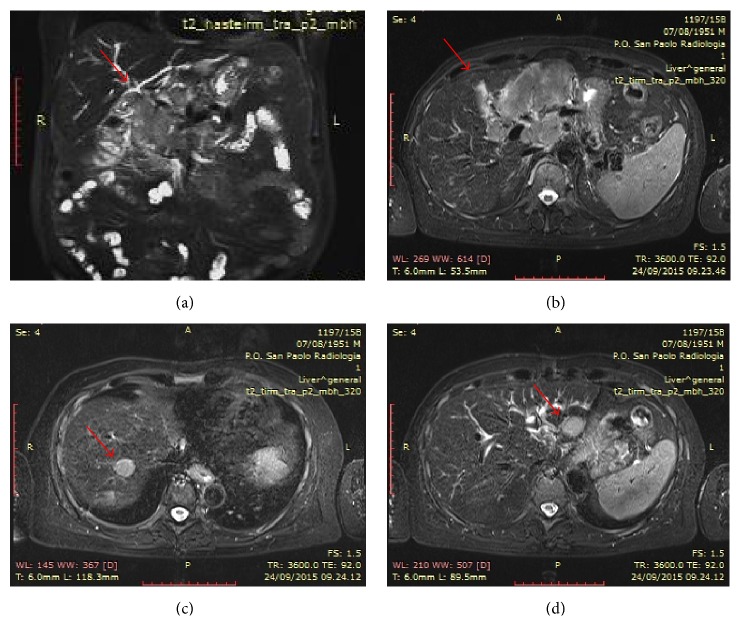
MR cholangiopancreatography after VBD therapy. Panels (a) and (b) show progression of extramedullary disease in the primary site (increase of dimension) and panels (c) and (d) show the appearance of new lesions in the liver.

The evaluation after eight cycles of BPR (August/September 2015) showed a new serological response of the disease (CM: 13 g/L, Hb 119 g/L, albumin 34 g/L, normal free light chain ratio, and clonal bone marrow plasma cells 3%) but progression of extramedullary disease because of the increase of the dimension of the primary plasmocytoma (Figures 3(a) and 3(b)) and because of the appearance of new lesions in the liver (Figures 3(c) and 3(d)), at peritoneum and subcutaneous level.

Patient underwent salvage therapy with D-PACE (dexamethasone, cis-platinum, adriamycin, cyclophosphamide, and etoposide) and after two cycles of therapy (February 2016) obtained a good serological disease response and regression of extramedullary plasmacytoma. From March 2016 patient underwent continuous treatment with pomalidomide and dexamethasone.

## 3. Discussion

The clonal evolution theory has been refined to include the concepts of cancer stem cell and intermediate subclones with cancer stemness properties, the importance of genomic instability, the role of epigenetics, and the impact of cancer microenvironment on clonal selection [7, 8]. In fact, the Darwinian behavioural characteristics of cancer stem cells are applicable to MM. Following its initiation, the myeloma propagating cell, which should be considered as the unit of evolutionary selection in Darwinian terms [9], becomes heterogeneous, allowing the transition from a normal plasma cell to the final stage of the transformation process, plasma cell leukaemia (PCL) or extramedullary myeloma [7]. MM cells presented with a significant number of karyotypic aberrations, suggesting that genomic instability plays a major role in MM clonal heterogeneity and evolution [10].

Moreover, growing evidence supports a pivotal role of the microenvironment in guiding clonal evolution and heterogeneity in MM [11, 12]. MM cells and mesenchymal/stromal cells spatially interact via adhesion molecules, communicate by cytokines and growth factors, and exchange macromolecules such as nucleic acid and proteins via microvesicles and exosomes with subsequent bidirectional signalling, typically resulting in a survival advantage for MM cells [13–15]. Finally, clinical and biological findings indicate that the disappearance and dominance of different clones in MM patient appeared to be determined also by selective pressure from treatment [16].

Our case is indicative of the selection of almost two different clones in this patient with different biological and clinical behaviour. The different response to therapy of medullary and extramedullary disease indicate that, immediately after ASCT, under the pressure of therapy, an aggressive and refractory clone has been selected. This clone, resistant to new drugs, rapidly spread at extramedullary sites and progressed under proteasome inhibitors and immunomodulatory therapy. Only an aggressive treatment induced a response of both clones with remission of bone marrow disease and extramedullary spread.

The knowledge that intraclonal heterogeneity is an important feature of MM biology has changed our way of addressing cancer, now considered as a composite mixture of clones and not as a linear evolving disease. In this variable therapeutic landscape, it is of primary importance for clinicians and researchers to consider the impact that evolutionary biology and intraclonal heterogeneity have on the treatment of myeloma and the emergence of resistance clones. Only by doing so will we be able to effectively use all of the new tools we have at our disposal to cure myeloma and to use treatment in the most effective way possible. All this evidence provides a strong biologic rationale for using combinatory chemotherapy in an attempt to eradicate all clones and avoid selection of aggressive ones.
